# Interventional Microbubble Enhanced Sonothrombolysis on Left Ventricular Assist Devices

**DOI:** 10.1002/advs.202201291

**Published:** 2022-05-26

**Authors:** Xiaobing Zheng, Yunfan Pan, Yuan Zhang, Kuilin Meng, Jianye Zhou, Xin Wang, Yongchun Cui, Jiang Li, Yongjian Li, Haosheng Chen

**Affiliations:** ^1^ State Key Laboratory of Tribology Department of Mechanical Engineering Tsinghua University Beijing 100084 China; ^2^ School of Mechanical Engineering University of Science and Technology Beijing Beijing 100083 China; ^3^ Animal Experiment Center Fuwai Hospital Chinese Academy of Medical Sciences Beijing 100037 China

**Keywords:** interventional therapy, LVAD, microbubble, thrombolysis, ultrasound

## Abstract

The left ventricular assist device (LVAD) is often used in the treatment of heart failure. However, 4% to 9% implanted LVAD will have thrombosis problem in one year, which is fatal to the patient's life. In this work, an interventional sonothrombolysis (IST) method is developed to realize the thrombolysis on LVAD. A pair of ultrasound transducer rings is installed on the shell of LVAD, and drug‐loaded microbubbles are injected into the LVAD through the interventional method. The microbubbles are adhere on the thrombus with the coated thrombus‐targeted drugs, and the thrombolytic drugs carried by the bubbles are brought into the thrombus by the cavitation of bubbles under the ultrasound. In a proof‐of‐concept experiment in a live sheep model, the thrombus on LVAD is dissolved in 30 min, without damages on LVADs and organs. This IST exhibits to be more efficient and safer compared with other thrombolysis methods on LVAD.

## Graphical abstract

An interventional sonothrombolysis (IST) method is developed to realize high efficient and safe thrombolysis on LVAD with integrated ultrasound units and thrombus‐targeted drug‐loaded microbubbles. It is verified by proof‐of‐concept experiments on sheep that the thrombus can be dissolved in 30 min without damage on LVADs and organs. This method has great potential to solve the fatal thrombosis problem of LVAD for patients with heart failure.

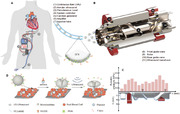

## 1 Introduction

In recent years, the left ventricular assist device (LVAD) has been widely used in the clinical treatment of patients with heart failure. However, pump thrombosis is always a severe complication, with an incidence of 4% to 9% events per patient‐year.^[^ ^1^ ^]^ Once the incidence occurs, the LVAD fails and the patient's life is in danger. Although there are some thrombolytic therapies in trials,^[^ ^2^, ^3^ ^]^ an efficient and safe treatment is still an urgent demand for the LVAD thrombosis in clinical practice.

Currently, the most common therapy on LVAD thrombus is medical therapy. However, it takes relative long time and has a high risk of intracranial hemorrhage.^[^ ^2^ ^]^ Alternatively, the pump can be replaced through an open‐heart surgery,^[^ ^3^ ^]^ but it is not proper for the patients because they are usually too weak to suffer another open‐heart surgery. To treat the LVAD thrombosis, we consider to adopt the interventional sonothrombolysis (IST), such as EKOS, which was approved by FDA in 2004, and it is used for the treatment of pulmonary embolism (PE), deep vein thrombosis (DVT) and arterial occlusion.^[^ ^4^ ^]^ In the sonothrombolysis therapy, an ultrasound field is generated on the thrombus, and the thrombolytic drugs and ultrasound contrast agents (e.g., microbubbles, nanodroplets, and magnetic microbubbles) are injected into the vascular at the upstream of the thrombus. The thrombus will be dissolved under the joint effect of the ultrasound and the thrombolytic drugs.^[^ ^5^, ^6^, ^7^, ^8^ ^]^ The interventional therapy reduces the doses of the thrombolytic agents to avoid the hemorrhage, and it can be applied to most of the patients. However, IST has not been applied to the LVAD nowadays because of two challenges: i) The ultrasound field is difficult to be generated in the LVAD. The rotating rotor of the pump prevents the probe of the ultrasound transducer to be placed into the LVAD, and the percutaneous ultrasound is difficult to reach LVAD. ii) The local concentration of the thrombolytic agent in LVAD is low. When the thrombolytic agent is injected into the LVAD, they will be flushed away in a short time.

Here, the two challenges are overcome and the IST is applied to the LVAD. A pair of ultrasound transducer rings is installed on the shell of the LVAD to generate an ultrasound field inside the pump, and drug‐loaded microbubbles are introduced to the pump by intervention. The microbubbles will adhere on the thrombus through the thrombus‐targeted peptide, coated on their surface, and the microbubbles will collapse and release the carried thrombolytic agents to dissolve the thrombus under the ultrasound field. Proof‐of‐concept experiments performed on sheep have shown that the microbubbles enhanced IST is an effective and safe therapy method to treat the LVAD thrombosis.

## 2 Results

### 2.1 Interventional Sonothrombolysis on LVAD

**Figure 1:**
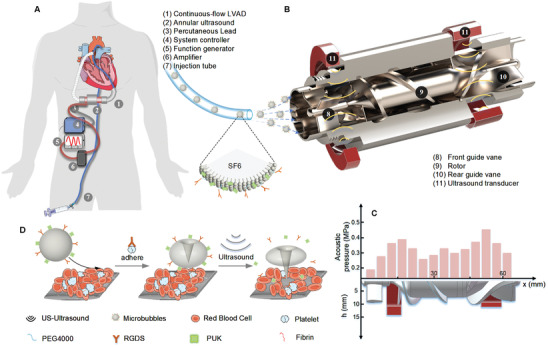
The schematics of IST in LVAD. A) Illustration of the interventional strategy of IST in LVAD. B) The strategy of IST with drug‐loaded microbubbles in the treatment of thrombus in axial flow pump. C) The generated ultrasound field inside the LVAD. D) The mechanism of the enhanced thrombolysis by the mechanical impaction from the collapse of microbubbles in the ultrasound field.

Microbubbles are introduced in front of the front vane of LVAD through a catheter along the descending aorta, as shown by the blue line in **Figure** **1**A. The microbubbles carry thrombolytic agents of pro‐urokinase (PUK) and thrombus‐targeted peptide of the arginine‐glycin‐aspartate‐serine (RGDS) on their shell, and the RGDS will adhere the bubbles on the thrombus in the LVAD.^[^ ^9^ ^]^ This drug‐loaded microbubble will increase the local concentration of thrombolytic agents in LVAD. The details on the drug‐loaded bubbles preparation are provided in Experimental Section, and the confocal images of the drugs on the bubble surfaces are shown in(Figure S1, Supporting Information).

The LVAD used in this work is an axial flow pump. To generate an ultrasound field in the LVAD, a pair of ultrasound transducer rings is installed on the metal shell of the pump around the front and the rear guide vanes, as shown in Figure 1B. The frequency of the ultrasound is 1 MHz, and the mechanical index (MI) is 0.5, which is within the safe range of the medical ultrasound standard.^[^ ^10^ ^]^ When the ultrasound transducers work, the ultrasound field is formed inside the pump throughout the front vane and the rear vane, which is measured by a needle hydrophone (NCS‐1, Institute of Acoustics of the Chinese Academy of Sciences), as shown in Figure 1C. The details on the ultrasound field measurement are provided in Experimental Section. Therefore, the ultrasound field is generated in the LVAD, without preventing the running of the LVAD.

When microbubbles flow into the pump, some of them will adhere on the thrombus due to peptides coated on their shell. The ultrasound will cause cavitation of the adhered microbubbles, and the mechanical impaction including the microjet and the shockwave will generate.^[^ ^11^, ^12^, ^13^ ^]^ The mechanical impaction will loosen the thrombus and bring the thrombolytic agents into the thrombus to accelerate the thrombolysis process, as shown in Figure 1D.

### 2.2 In Vivo Experiment of Sonothrombolysis

**Figure 2:**
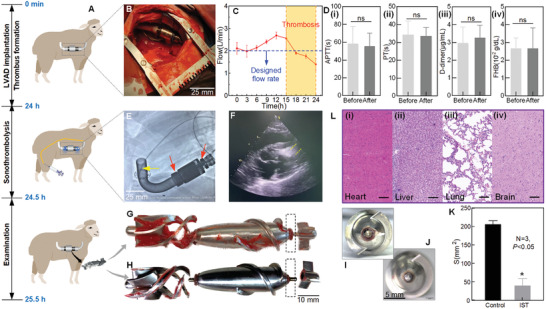
The in vivo experiments of IST. A) The treatment procedures of a proof‐of‐concept experiment on sheep. B) LVAD with a pair of ultrasound rings is implanted into the sheep. C) The flow rate of LVAD after implantation. D) Monitoring map of hemolysis and coagulation indexes of sheep model before and after LVAD thrombolysis. (*n* = 3; *P < 0.05). E) The DSA (Digital Subtraction angiography) image of the interventional catheter (yellow arrows) inserted in front of the pump. Red arrows: a pair of ultrasound transducer rings. F) The Echocardiogram image of microbubble injection. G) The thrombi on the pumps of the control group. H) The thrombi on the pumps of the IST experimental group. I) and J) are the thrombi on the bearing of rotor in the control group and in the experimental group, respectively. K) Statistical results of thrombus area in the control and in the IST therapy group. L) Histological analysis of major organs (heart, liver, lung, and brain) after interventional sonothrombolysis (IST). Scale bar: 100 µm.

The IST technology is verified in a proof‐of‐concept experiment. The experiment includes three main stages as shown in **Figure** **2**A: i) LVAD implantation, ii) Sonothrombolysis, and iii) Examination. In stage i), a total of five sheep are randomly assigned to the treatment group (three sheep) or the control group (two sheep). Each sheep is implanted with a LVAD (Figure 2B). In order to form the thrombus in the LVAD in a short time, ACT (Activated Clotting Time of whole blood) is maintained at 160–200 s by heparin after the LVAD implantation, which is usually >200 s on the assist device in the in‐vivo experiment.^[^ ^14^ ^]^ After 15 h, the flow rate of the heart pump is observed to decrease continuously and the flow rate decreases by >20% after 24 h compared with the designed value 2 L min^−1^ of the pump, as shown in Figure 2C. It indicates that the thrombus has been formed in LVAD.

In stage ii), sonothrombolysis is performed in the experimental group. The microbubbles with the concentration of 3.27 × 10^7^ MBs mL^−1^ are injected to the pump at a speed of 1 mL min^−1^ for every 5 min, and totally 12 mL bubbles are injected in 30 min through an interventional catheter. The outlet of the catheter is placed in front of the heart pump, as marked by the yellow arrow in Figure 2E. The injection of PUK‐loaded microbubbles is observed by Echocardiogram, as shown in Figure 2F, and the video of the microbubbles sprayed in the heart is provided in Movie S1, Supporting Information. During the whole injection process of microbubbles, the ultrasound is kept working.

In stage iii), the LVADs in the experimental group and in the control group are all taken out and examined. On the LVAD of control group, thrombi are obvious and its average area is 206.0 mm^2^ as shown in Figure 2G. In the experimental group, only small area is covered by thrombus as shown in Figure 2H. The average area of the thrombus on the rotor is 40.8 mm^2^, and the thrombolysis results are shown in Figure 2K. More than 80% thrombus on the rotor is dissolved. The thrombus on the bearing usually increases the friction to cause the failure of the axial flow pump. The volume of the thrombus at the bearing of the front guide vane is 24.9 mm^3^ in the control group, as shown in Figure 2I, and the volume of the thrombus at the bearing of the leading vane is 5.0 mm^3^ in the experimental group, as shown in Figure 2J. The images of the thrombus on all the LVADs after the experiments are provided in (Figure S2, Supporting Information). According to the result, the thrombolytic efficiency of microbubbles enhanced IST is >80% in 30 min, which is much faster than other current thrombolysis therapies.^[^ ^2^ ^]^

The safety of this IST method is also assessed. The MI of the ultrasound is controlled to be <1.0, which is within the safe range according to the IEC standard 61157–2007.^[^ ^10^ ^]^ In the experiments, hemolysis and coagulation are monitored before and after IST treatment, as shown in Figure 2D. There is no significant difference of the four indicators before and after the treatment, which indicates that the treatment has a good biocompatibility. The hematoxylin and eosin (H&E) staining results of the main organs of the sheep are shown in Figure 2L, where no histological toxicity and no inflammatory lesion are found. Also, no large debris is found to block the blood vessels. Therefore, the microbubble enhanced sonothrombolysis is a safe treatment for the LVAD.

### 2.3 In‐Vitro Study of Sonothrombolysis

**Figure 3:**
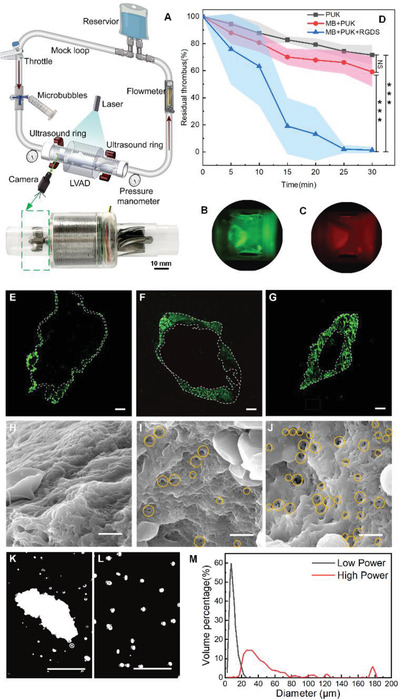
The in vitro studies on the thrombolytic mechanism of IST. A) The mock loop device for thrombolysis in vitro contains a visual segment for observing the thrombus on the LVAD. B) The thrombus growing on the front vane dyed in green fluorescence. C) The microbubble stained with red fluorescence adhere on the thrombus. D) Residual thrombus on the LVAD in group (1)–(3). (*n* = 3; ***P < 0.001). E–G) Confocal images of drug penetration into the thrombus at 15 min of the three groups. Scale bar: 200 µm. H–J) SEM images of sono‐porosity effect on thrombus during thrombolysis at 15 min of the three groups. Yellow circles: the location of the holes. Scale bar: 2.5 µm. K) Thrombus debris of higher power and L) regular power was observed under light microscope. Scale bar: 100 µm. M) The statistics results of thrombus debris under different ultrasound power.

To investigate the mechanism of microbubbles enhanced sonothrombolysis, in vitro experiments are performed in a mock loop. The mock loop is designed according to ASTM standard F1841‐97 (R 2017), as shown in **Figure** **3**A. The LVAD installed in the mock loop has a transparent shell made of polymethyl methacrylate (PMMA) for observing the thrombolytic process. Sodium citrate anticoagulated porcine blood is running in the channel at the flow rate of 2 L min^−1^, and 5 mL of 0.2 M calcium chloride solution is then injected to the channel in front of the LVAD at a rate of 0.1 mL min^−1^ as a coagulant for clotting. The Sodium citrate anticoagulated porcine whole blood is mixed with the green fluorescent reagent fluorescein FITC (F1906, Thermo Fisher) and DiOC_6_ (D273, Thermo Fisher) to label platelets and proteins for 15 min before it is injected into the mock loop. After 1 h, the flow rate decreases by >20%, and the thrombus is formed on the LVAD. After the thrombus is formed, the whole blood is replaced by the blood plasma so that the thrombolytic process on the front vane of the LVAD can be seen through the transparent shell. The area of thrombus (in green color) on the front vane is recorded using a fluorescent camera, as shown in Figure 3B. When the microbubbles adhere on the thrombus, the 5‐TAMRA (5‐Carboxytetramethylrhodamine) on the bubbles will show red fluorescence, as shown in Figure 3C, which illustrates that the bubbles can adhere on the thrombus even in the fast flowing blood. Moreover, the adhesion of microbubbles to the thrombus gradually increased with time under the fast flow conditions, as shown in Figure S3, Supporting Information.

In the in vitro experiment, there are three groups of sonothrombolysis to investigate the enhancement of microbubbles: (1) PUK (2917 U mL^−1^, consistent with the amount of PUK loaded in each milliliter of microbubbles) is injected to the plasma at a rate of 1 mL min^−1^ for every 5 min, and totally 12 mL are injected in 30 min, (2) microbubbles with PUK but without RGDS are added in the plasma and they are injected at a rate same to group (1), (3) microbubbles with PUK and RGDS are added in the plasma with the same injection rate as the group (1). The thrombolysis process has been recorded every 5 min. The thrombus residual *S* is calculated according to *S* = *S*_US_/S_0_ × 100%, where *S*_0_ is the original area of the thrombus and *S*_US_ is the remaining area of the thrombus. It's obvious that group (3) has the highest thrombolytic efficiency, as shown in Figure 3D.

The enhanced thrombolysis effect of microbubbles is attributed to the sono‐porosity mechanism.^[^ ^15^ ^]^ The distribution of PUK inside the thrombus during the sonothrombolysis in the group (1) and (2) are compared with group (3). PUK only stays at the edges of the thrombus in group (1) (2), as shown in Figure 3E,F, while in group (3), the PUK diffuse into the inner of the thrombus, as shown in Figure 3G. The fluorescent dye method is introduced in the Experimental Section. Actually, much more holes are found on the surface of the thrombus in group (3) than in group (1) and group (2), as shown in Figure 3H–J and Figure S4, Supporting Information.

The sono‐porosity effect can be enhanced by increasing the ultrasound strength. However, the increase of the ultrasound power will result in larger debris of thrombus, as shown in Figure S5, Supporting Information. We compare the size distribution of debris under the ultrasound power of 1.3 and 6.0 W as shown in Figure 3M, and the images of the debris in the two conditions are shown in Figure 3K,L, respectively. The thrombus debris produced by the power 1.3 W are <20 µm, while 200 µm debris appear in the power of 6.0 W. Even at high power ultrasound, the maximum particle size of thrombus debris produced is below 500 µm. The particle size analysis in our experiment matches well with the study by Goel et al.^[^ ^16^ ^]^ It should be noted that the effect of ultrasound in the sonothrombolysis is to enhance the diffusion of thrombolytic drug to dissolve the thrombus, not to break the thrombus by the mechanical impaction. Therefore, the power of the ultrasound used in IST therapy need to be controlled in the range, within which the thrombolytic efficiency is high, while large debris is not generated. In the in vivo experiment, the ultrasound power is 1.3 W, and it achieve a high efficiency while keep the safe of the therapy.

## 3 Discussion

**Figure 4:**
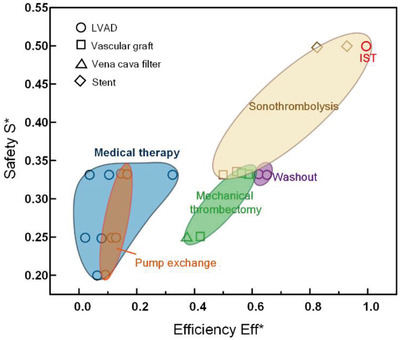
The safety and efficiency of IST therapy compared with other thrombolysis techniques for cardiovascular implantations.

It is the first time that we adopt the IST to treat the thrombosis on LVAD. Here, we review clinical studies about thrombolytic technologies on implantable and interventional devices in recent years ^[^ ^2^, ^3^, ^15^, ^16^, ^17^, ^18^, ^19^, ^20^, ^21^, ^22^, ^23^, ^24^, ^25^, ^26^, ^27^, ^28^, ^29^, ^30^, ^31^, ^32^, ^33^, ^34^, ^35^ ^]^ and compare the IST with other currently used thrombolytic technologies, including medical therapy, pump exchange, sonothrombolysis, washout maneuver, and mechanical thrombectomy. Safety and efficiency are focused on in the evaluation. The risk of each therapy has been scored, as shown in Table S1 in Supporting Information. The safety of the treatment is defined as S* = 1/risk score. The efficiency is defined as Eff* = 1/(risk score *× t*), where *t* is the treatment time. The safety and efficiency of the current thrombolysis methods are compared in **Figure** **4**. Medical therapy is usually the first choice to treat the LVAD thrombosis. However, it has the incidence of hemorrhagic stroke,^[^ ^2^, ^3^, ^15^, ^16^, ^17^, ^18^, ^19^, ^20^ ^]^ and need a relative long treatment time, usually >3 h.^[^ ^2^ ^]^ Pump exchange is an urgent treatment, but it has a high surgical risk because most of the patients can't suffer another reopen heart surgery.^[^ ^21^, ^22^, ^23^, ^24^, ^25^, ^26^ ^]^ Washout maneuver is used to remove the thrombus at the inlet of LVAD. Although it shows a good success ratio, the risk is still high because the thrombus washed from LVAD will enter other organs.^[^ ^3^, ^27^ ^]^ Mechanical thrombectomy is currently widely used in vascular graft thrombosis,^[^ ^28^, ^29^, ^30^, ^31^, ^32^ ^]^ but this therapy has a risk of damaging the inner wall of the blood vessels in the treatment site. Sonothrombolysis has been widely used in vein bypass grafts thrombolysis: the ultrasonic probe is inserted into the thrombus through intervention of the catheter.^[^ ^33^, ^34^, ^35^ ^]^ But this method cannot be directly adopted in the LVAD thrombolysis because the probe will collide with the moving rotors in LVAD. Compared with those methods, the IST technology in this work exhibits high efficiency and safety, which will have potential applications in clinical treatment.

However, the IST for the LVAD thrombolysis has some limitations. The IST needs thrombolytic drug‐loaded microbubbles to dissolve the thrombus, which bring the IST in the risk of hemorrhage in clinical treatment. When treated with thrombolytic agents, such as tissue plasminogen activator (tPA), long treatment times and high doses of tPA can greatly increase the risk of internal bleeding due to off‐target effects, and most notably intracranial bleeding that can lead to stroke or death.^[^ ^2^ ^]^ To solve this problem, the interventional drug delivery and the targeted microbubble can reduce the doses of the thrombolytic agents to avoid the hemorrhage. In the in vivo experiments, the thrombolytic agents carried by the bubbles for the thrombolytic treatment is totally 35 000 units, which is only 0.7% of the dose usually used in medical therapy.^[^ ^36^ ^]^ More experiments need to be performed to optimize the dose of the thrombolytic agents carried by the bubbles. Another question is on the long‐term effect of the therapy. The IST is currently for urgent therapy for the LVAD thrombus. It is effective to restore the LVAD in a short time. However, the recurrent thrombosis on LVAD is not examined, which needs a relatively long time. More in vivo experiments still need to be performed besides this proof‐of‐concept experiment. In addition, we have only tested the IST technology on axial flow pump, and in the future, the IST technology can be extended to centrifugal pump, which also accounts for a large proportion of clinical applications, by adjusting the shape of the ultrasonic transducer and re‐matching the ultrasonic field.

## 4 Conclusion

In this work, an IST method is developed to treat the thrombosis on LVAD. A pair of ultrasound transducer rings is installed on the shell of the LVAD to generate local ultrasound field inside the device. Then, drug‐loaded microbubbles are brought to the inlet of the LVAD through an interventional catheter. Under the ultrasound field, the bubbles collapse and enhance the diffusion of the thrombolytic drugs into the thrombus to accelerate the thrombolysis process. The thrombus targeted agents coated on microbubbles can adhere on the thrombus in the fast flowing blood, which increases the local concentration of microbubbles and improves the thrombolysis efficiency. The proof‐of‐concept experiments performed on sheep demonstrate that this method is effective and safe, and it is a promising therapy method in clinical treatment on LVAD thrombosis.

## 5 Experimental Section

### Materials

Experimental animal sheep, heparin, propofol, isoflurane, potassium chloride, and whole pig blood were provided by Fuwai Hospital, Chinese Academy of Medical Sciences. Whole blood was collected from healthy animals using a venipuncture and stored in blood bag containing sodium citrate to prevent coagulation. 3’,3’‐dihexyloxacarbocyanine iodide (DiOC6) and FITC labeled human fibrinogen were purchased from Thermo Fisher Scientific (Waltham, MA, USA). RGDS (# 0 401 001 3130) and RGDS‐5‐TAMRA (5‐Carboxytetramethylrhodamine) peptide were obtained from Jiangsu Qiangyao Biological Technology (Co. Ltd. Jiangsu, China). SonoVue (Bracco Suisse SA.) was a commercially available clinical ultrasound contrast agent. Recombinant Human Urokinase Pro (PUK, # 20201102) was purchased from Tasly Pharmaceutical (Co. Ltd. Shanghai, China). Ethanol was purchased from Sinopharm Chemical Reagent Co., Ltd. 10% formalin, 2.5% glutaraldehyde solution and Triton‐X 100 were from Beijing Solarbio Science & Technology Co., Ltd. Phosphate‐buffered saline (PBS, 1×) is obtained from HyClone. The primary antibodies anti‐uPA (# sc‐59727) was purchased from Santa Cruz Biotechnology. The goat antimouse Alexa Fluor 488‐conjugated (# ab150113) was from Abcam.

### Study Design

The purpose of this study was to investigate the safety and efficiency of the IST method to treat the thrombosis on LVAD. A pair of ultrasound transducer rings was installed on the shell of the LVAD to generate local ultrasound field inside the device. And drug‐loaded microbubbles were interventional introduced to the pump. The efficacy and safety were assessed by experiments on mock loop in the in vitro and animal experiments in vivo. According to the proof‐of‐concept experiment on sheep, the thrombolytic efficiency of this strategy for LVAD thrombosis can reach >80% within 30 min.

### Preparation of Drug‐Loaded Microbubbles

The drug‐loaded microbubbles are prepared by adding 125 µL of 10 mg mL^−1^ RGDS (Batch No. 04010013130, Jiangsu Qiangyao Biological Technology Co. Ltd. Jiangsu, China) and 1 mL of 500 000 U mL^−1^ PUK (Batch No. 20201102, Tasly Pharmaceutical Co. Ltd. Shanghai, China) to every 5 mL of microbubbles (SonoVue, Bracco Suisse SA). Then, the solution containing bubbles, PUK and RGDS was stirred in a mixer (DLAB, MX‐RD‐E) at the speed of 80 rpm for 10 min. Then, the solution is put in the centrifuge (MiniSpin, Eppendorf) and run for 2 min at 800 rpm. Take the microbubbles out and discard the subnatant. Finally, add saline solution (0.9% NaCl) to the solution till the volume reaches 12 mL. 5‐TAMRA (5‐Carboxytetramethylrhodamine) has been a widely used fluorophore for preparing bioconjugates, especially fluorescent antibody and avidin derivatives used in immunochemistry. RGDS‐5‐TAMRA peptide (Jiangsu Qiangyao Biological Technology Co. Ltd) replaces RGDS peptide when the adhesion of bubbles in the mock‐loop was observed. The microbubbles will be in red fluorescence when they are excited by the fluorescent light with a wavelength of 546 nm.

### Acoustic Field in LVAD

The transducer ring is composed of a stack of piezoelectric ceramic (PZT‐4) sheets. PZT‐4 sheets are molded into a strip transducer with ethylene propylene (EP) and then the strip transducer was bent to have a ring shape after being heated in the oven at 70 ℃. The rings were placed in the water tank filled with degassed water. The transducer ring was bonded to the hull of LVAD with EP and the 1.0 MHz ultrasound energy was delivered to the LVAD through the EP (acoustic matching layer) to the hull then to the blood in the LVAD. The ultrasound source was input by continuous sinusoidal wave at 1 MHz using an arbitrary function generator (AFG3021, Tektronix Inc., Beaverton, OR, USA) connected with a 12 dB radio‐frequency amplifier (FPA301, FeelElec Inc., China). A needle hydrophone (NCS‐1, The Institute of Acoustics of the Chinese Academy of Sciences) was used to measure the acoustic field in LVAD. During the measurement, the LVAD is not working and the acoustic pressure is measured every 5 mm along the axis of the LVAD. No matter whether the LVAD was working or not, the maximum ultrasound pressure output does not vary significantly, as shown in Figure S6, Supporting Information.

The input parameters for thrombus debris evaluation by the power 1.3 W were 1 MHz excitation frequency, 36.6 Vpp input voltage, 100% duty cycle, and 1 MHz pulse repetition frequency. The input parameters for thrombus debris evaluation by the power 6.0 W are 1 MHz excitation frequency, 80 Vpp input voltage, 100% duty cycle, and 1 MHz pulse repetition frequency. The measurement of the corresponding ultrasound pressure output in the middle of ring for different powers used in thrombus debris analysis was plotted in Figure S7, Supporting Information.

### Examination after the Animal Experiment

After sonothrombolysis, animals are sacrificed with potassium chloride. The animal's heart and the heart pump were removed. The heart pump was disassembled and the thrombi on LVAD were measured. The tissues of lung, heart, liver, and brain were fixed in 10% formalin solution for histological analysis and qualified by H&E staining. The slides of the tissues were scanned on 3D HISTECH Pannoramic SCAN (3D Histech Company, Hungary) to obtain photos.

### In Vitro Experiment on Sonothrombolysis in the Mock Loop

The closed mock loop contains a polyvinylchloride (PVC) tubing, a reservoir, a sampling port, an electromagnetic flow probe and its corresponding flowmeter, a water bath, LVAD, throttle and pressure manometer (inlet and outlet of LVAD). The loop should be filled with saline solution that was recirculated for ≈10 to 20 min to rinse and wet all the blood‐contacting surfaces before experiment. The saline solution was drained completely from the loop prior to filling it with blood. After being washed with saline solution, the circuit was primed with 200 ± 50 mL of fresh blood into the reservoir bag. Air collected in the reservoir should be eliminated and no air interface left in the reservoir. The pressure monitoring was incorporated into the LVAD both at the inlet and outlet tubes. An electromagnetic flow probe was placed at the outlet side of the pump to monitor the flow rate. The flow rate of the LVAD is maintained between 2–5 L min^−1^ and the pressure was maintained at 100 mm Hg. These were the physiological conditions under which the LVAD was designed to be used. the differential pressure (*H*) versus flow rate (*Q*) relationships (*H–Q* curves) of the experimental transparent LVAD in the mock loop was measured and provided the results in Figure S8, Supporting Information. In the mock loop in the in vitro test, the LVAD inlet pressure was 5–8 kPa, and the outlet pressure was 13–19 kPa. These were the physiological conditions under which the LVAD was designed to be applied.

The relationship between ultrasound power and clot debris was measured by particle size analysis of the fluid in the mock loop after the sonothrombolysis experiment. The solutions were analyzed to particle imaging and size analysis using automatic image method particle size analyzer (W‐2000, Beijing Hangxin Tong Technology Co.). The morphology of the thrombus debris was also observed using a microscopy (IX83, Olympus Co.).

### Sono‐Porosity of the Thrombus

The thrombi on the LVAD in mock loop were sampled for the following studies in 15 min sonothrombolysis. The thrombi from each group were immediately fixed with 2.5% glutaraldehyde solution at 4 °C for 24 h after therapy. They were subsequently dehydrated with ethanol, dried at room temperature, and plate to observe the morphology of the thrombus. High‐definition micrographs were obtained from different randomly chosen areas of each thrombus or embolus to in a high‐resolution field emission scanning electron microscope (Zeiss). The porosity of multiple SEM images (*n* = 5) from group (1)–(3) was calculated using ImageJ. The porosity was expressed as a percentage of the pore area to the total area of the thrombus.

### Diffusion of Drugs In Thrombus

The thrombi, which experience 15 min IST, were fixed in 4% paraformaldehyde for 24 h. The fixed thrombus was embedded in paraffin and then sectioned and dewaxed. All procedures were performed in strict accordance with the tissue section preparation specifications. The histology sections are made along the sagittal plane with a thickness of 8 µm. After blocking with goat serum containing 0.3% Triton‐X 100 for 1 h at room temperature, the slides were incubated with primary antibodies overnight at 4 °C. After washing three times with PBS, the slides are incubated with fluorescence‐labeled secondary antibodies for 1 h at room temperature in the dark. Slices are washed three times in PBS, and then counterstained with Fluorescent mounting medium (ZLI‐9556; ZSGB‐Bio). The primary antibodies are as follows: anti‐uPA (1:00; sc‐59727, Santa Cruz Biotechnology). The secondary antibodies are conjugated to goat anti‐mouse Alexa Fluor 488‐conjugated (1:400; ab150113, Abcam). Fluorescence of PUK is observed under a Laser Confocal Microscope Nikon A1 (Nikon, Japan).

### Protocol of Animal Experiment

The animal ethics committee of Fuwai Hospital, Chinese Academy of Medical Sciences, approved all animal experiments and procedures. All animal research strictly abides by the regulations on animal protection of Fuwai Hospital. The protocol of surgery on sheep was provided in Supplementary Methods.

### Statistical Analysis

All experimental data were expressed as mean values ± SD and are analyzed using SPSS 19.0 (SPSS). Intergroup comparisons and analysis in each experiment are performed by unpaired Student's *t*‐test. Probability *(P*) values of <0.05 were considered statistically significant.

## Conflict of Interest

The authors declare no conflict of interest.

## Author Contributions

X.Z. and Y.P. contributed equally to this work. X. Zheng, Y. Pan, Y. Zhang, and K. Meng performed the experiments. Y. Li, J. Li, and H. Chen conceived and designed the study. J. Zhou, X. Wang, and Y. Cui did animal experiments. X. Zheng, Y. Pan, Y. Li, and H. Chen wrote the paper. Y. Li and H. Chen are both corresponding authors.

## Supporting information

Supporting Information

Supplemental Movie 1

## Data Availability

Research data are not shared.
