# Supplementary material for: Interventional Microbubble Enhanced Sonothrombolysis on Left Ventricular Assist Devices
Source: Adv Sci (Weinh). 2022 May 26;9(21):2201291. doi: 10.1002/advs.202201291 (PMC9313509; doi:10.1002/advs.202201291)
Supplement: Supplementary file 1 — Supporting Information [file ADVS-9-2201291-s002.pdf]

Supporting Information

Interventional microbubble enhanced sonothrombolysis on left ventricular assist devices

Xiaobing Zheng, Yunfan Pan, Yuan Zhang, Kiulin Meng, Jianye Zhou, Xin Wang, Yongchun Cui, Jiang Li, Yongjian Li, and Haosheng Chen**

X. Zheng, Y. Pan, K. Meng, Y. Li, H. Chen
State Key Laboratory of Tribology
Department of Mechanical Engineering
Tsinghua University
Beijing 100084, China
E-mail: liyongjian@tsinghua.edu.cn; chenhs@tsinghua.edu.cn

Y. Zhang, J. Li
School of Mechanical Engineering
University of Science and Technology Beijing
Beijing 100083, China

J. Zhou, X. Wang, Y. Cui
Animal Experiment Center
Fuwai Hospital
Chinese Academy of Medical Sciences
Beijing 100037, China.

Author contributions:

X. Zheng and Y. Pan contributed equally to this work. X. Zheng, Y. Pan, Y. Zhang and K. Meng performed the experiments. Y. Li, J. Li and H. Chen conceived and designed the study. J. Zhou, X. Wang and Y. Cui did animal experiments. X. Zheng, Y. Pan, Y. Li and H. Chen wrote the paper. Y. Li and H. Chen are both corresponding authors.

Supporting Methods:

The protocol of animal experiment: The animals are given heparin intravenously and the activated blood clotting time (ACT) is maintained at 160-200 s when propofol-induced

anesthesia is successfully performed. Endotracheal intubation is performed, and isoflurane is inhaled for general anesthesia maintenance. For the two groups, the input of LVAD type axial flow circulation auxiliary pump is inserted into the left ventricle through the apex of heart. Then the output is end-to-side anastomosed to descending aorta, and the reliability of connection are confirmed. Then, the axial flow pump is turned on, and the flow rate is set closely to the preoperative cardiac output. The Swan-Ganz catheter is placed in the heart for cardiac output and pulmonary artery wedge pressure observation, and the success of unloading is confirmed. The speed of blood pump is 8000 rpm, and the flow rate maintains at around 2.0 L/min. Negative pressure drainage is placed in left thoracic cavity. The thoracic cavity is closed in the routine process.

The animals installed with the heart pump combined with ultrasound transducer closely monitor blood pressure, heart rate, body temperature, ACT, pump speed, flow, and current. When it is time to start to sonothrombolysis. The 5F pigtail catheter, a conventional subcutaneous tunnel (locked arterial sheath), has been chosen to establish microbubbles path from the femoral artery. And tip of the catheter injecting of microbubbles is 0.7-1.2 cm from the opening of the heart pump. During sonothrombolysis, the ultrasound probe continues to turn on in the heart pump.

Supporting Figures:

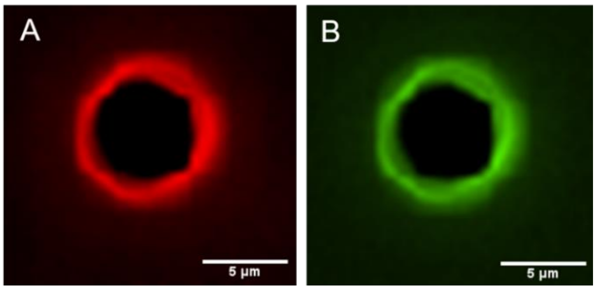

Figure S1. The structure of microbubbles. The microbubbles carry the thrombus-targeted drugs (RGDS) (A) and thrombolytic drugs (PUK) (B) on their surfaces.

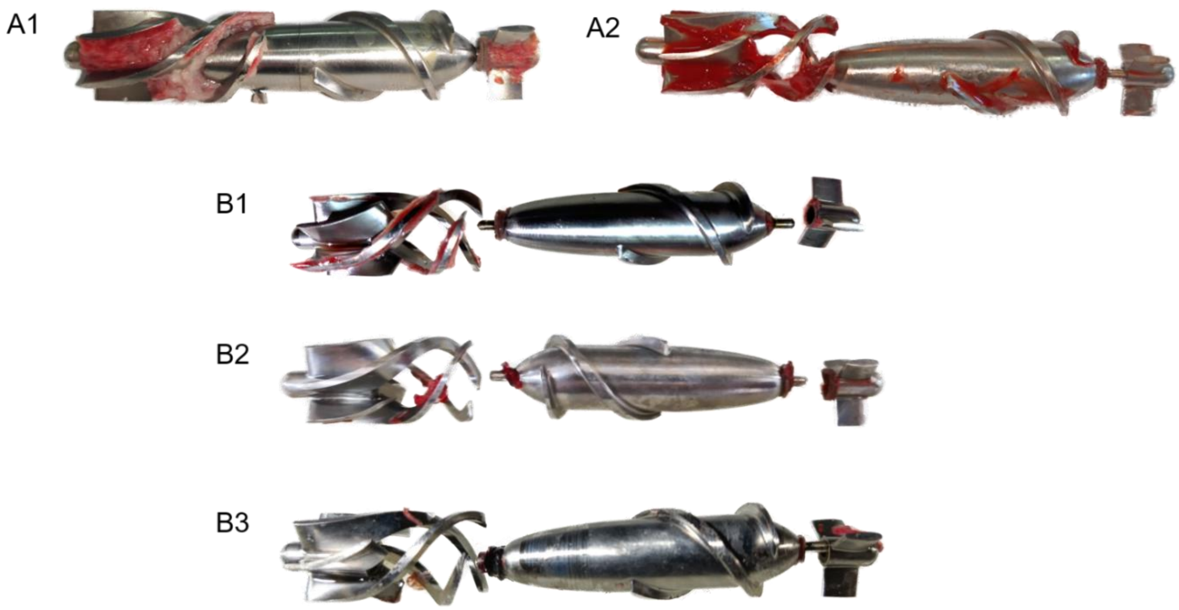

Figure S2. The images of the thrombi on all the LVADs after the experiments. (A) The thrombus on the LVADs of all the control groups. (B) The thrombus on the LVADs of all the IST experiment groups.

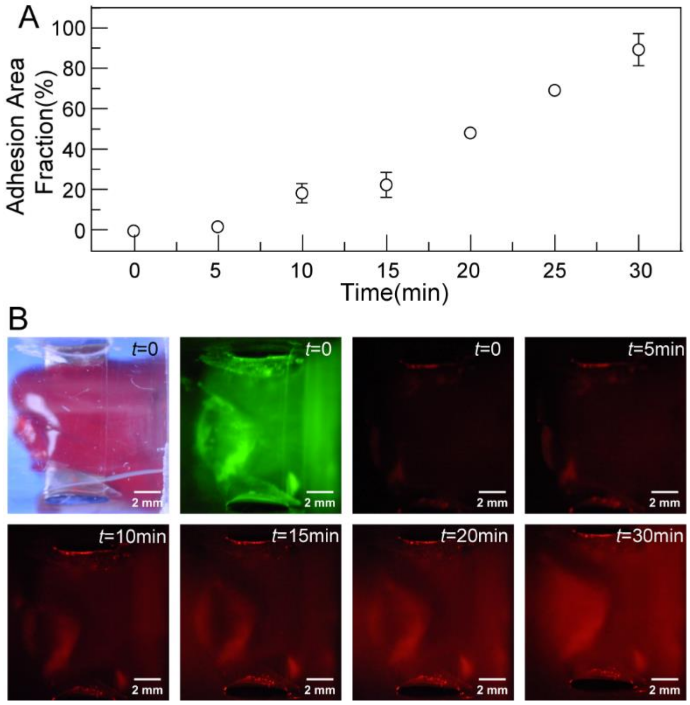

Figure S3. SonoVue MBs are targeted with RGDS, and then infused in front of the LVAD in mock loop at a clinical flow rate. (A) Variation of the number of adhered MBs with time. The area fraction of the adhered MBs is used. (B) Time sequence images of adhered MBs on the LVAD thrombus in targeted MBs group. Scale bars are 2 mm.

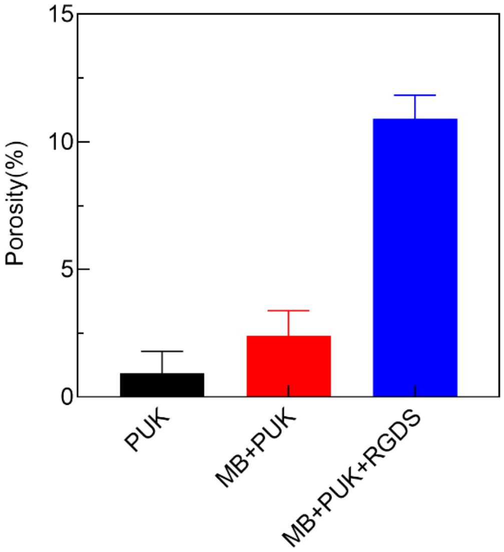

Figure S4. The porosity of the thrombi in group (1)-(3), calculated by SEM images (n=5).

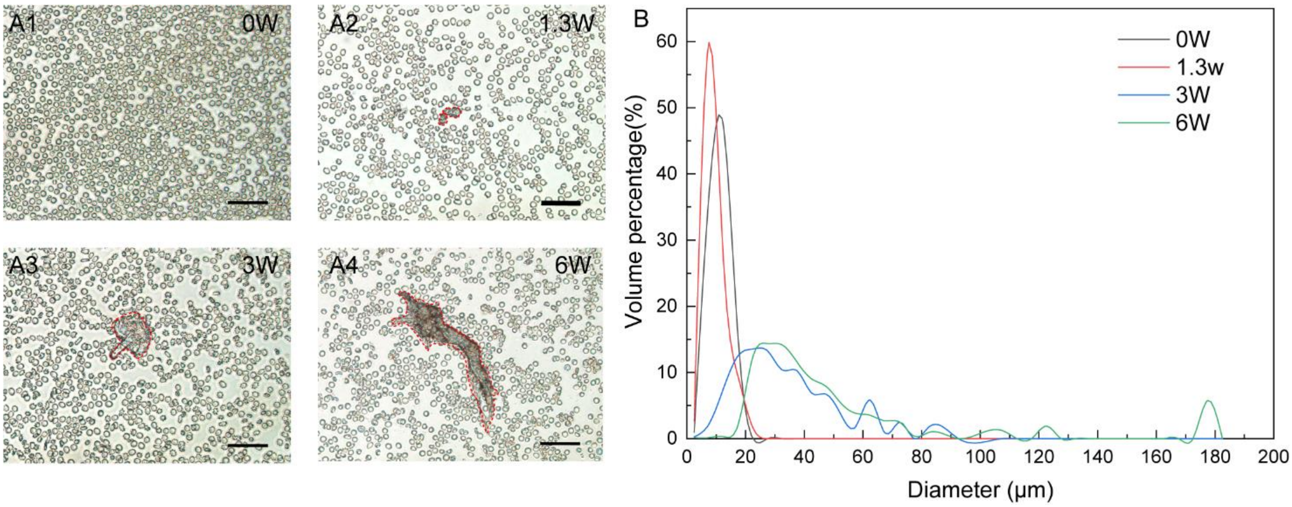

Figure S5. The morphology (A) and particle size (B) of thrombus debris changes with ultrasound power. Scale bars are 50 μm. (Drug and targeted-microbubbles are added in all groups, differing only in ultrasound power)

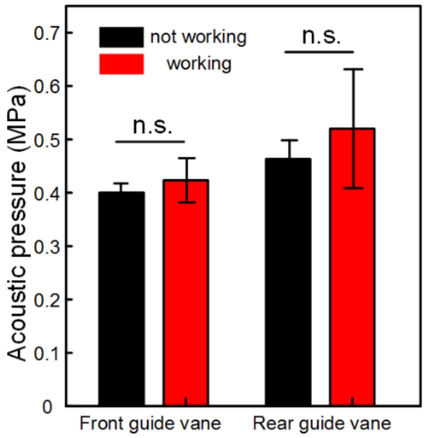

Figure S6. The measurement of maximum ultrasound pressure output at the front guide vane and rear guide vane when the LVAD is working or not. (n=3)

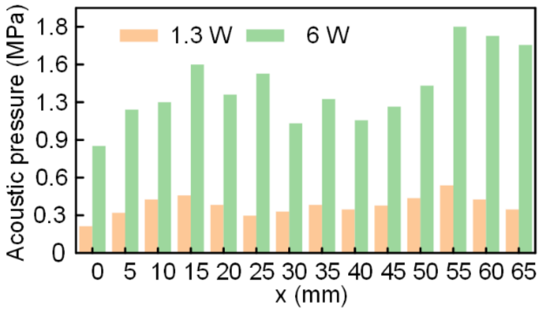

Figure S7. The measurement of the corresponding ultrasound pressure field inside the pump for 1.3 W and 6 W.

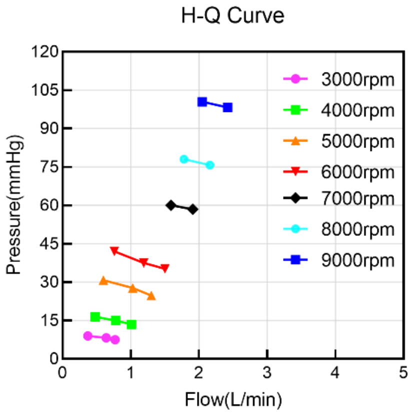

Figure S8. The differential pressure (H) versus flow rate (Q) relationships (H-Q curves) of the experimental transparent LVAD in the mock loop.

Supporting Table:

Table S1. Risk score (0-10 points) of thrombolysis techniques for LVAD and other implants.

Category		Points
Bleeding	None	0
	<20%	1
	≥20%	2
Obstruct	None	0
	Yes	1
Surgery	None	0
	Minimally	1
	Thoracotomy (≥50%)	2
Re-thrombosis	<30%	0
	≥30%	1
Adjuvant thrombolytic therapy	Yes	0
	No	1
Mortality	<10%	0
	≥10%	1

Supporting Movie:

Movie S1. The Echocardiography video of microbubble injection. The microbubbles are injected to the pump through an interventional catheter, and the outlet of the catheter is placed in front of the heart pump.
